# Modern Treatment of Cancer

**Published:** 1903-06-06

**Authors:** 


### MODERN TREATMENT OF CANCER.

Perhaps it has never been more necessary than it is at the present time, when the general public is directing its attention so closely to the scientific results of the various researches into the nature of malignant disease, to issue warnings lest exaggerated importance should be ascribed to methods of treating cancer which have not borne the test of time and experience. Whatever cancer is, in spite of all the attention paid to the subject, its true cause and nature remain shrouded in mystery. The best working hypothesis, upon which modern treatment is based, be it true or false, is that cancer is essentially, in its earliest stages, a local disease; and the treatment embraces remedies designed not only to prolong life indefinitely by the removal of the disease when in its primary stages, but also to support in the broadest-minded sense the doctrines of euthanasia. Although modern surgery is not the barbarous practice it was in ancient times, there is still, unfortunately, sufficient reason for the apprehension with which the average patient regards all cutting operations, and for this reason if for none other any method of treatment which could fairly claim to be as efficient as the surgeon's knife would indeed be welcome; but until that consummation is reached, it cannot be too strongly insisted that the most reliable treatment is to remove malignant disease early and freely, without losing valuable time in trying measures which at the best are uncertain and are probably useless. For endeavours to cure by means other than early and wide excision, have so far been almost invariably futile. Numbers of supposed "cancer cures" have been put forward from year to year, but every single one has failed to stand the test of time. Among them perhaps the most remarkable is that suggested by Coley. But the success of the treatment of cancer by Coley's fluid is very limited, and moreover its application is attended by so much danger, that from the direct effect of the treatment alone, some 10 per cent. cases prove fatal. In carcinoma of all forms, it is useless, and, with the single exception of the spindle-celled variety, the same is true of all forms of sarcomata. In fact, the success of Coley's fluid is limited to less than half the cases of spindle-celled sarcomata, and the method has now been practically discarded. So with the other "cancer cures" such as Beatson's method of oöphorectomy for mammary cancer, and treatment by thyroid extract. They seem to have effected a certain amount of improvement and even arrest of growth in some instances, but they cannot be claimed as "cures." At the present moment the various methods of treatment by light and electricity are being vaunted with considerable enthusiasm, and certainly it appears that good results have been achieved in a few cases, chiefly of small and superficially situated cancers, inasmuch as the tumours have disappeared. But the methods have not been in use sufficiently long to provide anything like reliable data on which to prognosticate the results which are likely to be obtained in any given case. And in more than one instance where a malignant ulcer has healed under the administration of $x$-rays, and been recorded as a cure, it has subsequently broken down again and extended rapidly without being in the least checked by further $x$-ray treatment. It is well in the meantime to seriously consider what may be the effect of premature conclusions when they are put forward as if they were established facts. It has probably fallen to the lot of more than one practising surgeon to be consulted by a patient who has shown himself to be suffering from cancer for a considerable time, but has deferred submitting himself to a radical operation in the hope of obtaining a cure by one of the many methods of light treatment, and after wasting much valuable time over such tentative treatment he turns to the surgeon too late. The opportunity for complete removal has been lost, and the patient has thrown away his only chance of recovery or relief. It is for this reason that it is advisable to warn the public from accepting too readily any account of a "cancer cure" which has not stood the tests of time and experience.

